# Müllerian Adenosarcoma: A Single-Centre Experience of 59 Cases of This Rare Entity

**DOI:** 10.7759/cureus.13360

**Published:** 2021-02-15

**Authors:** Iftikhar Ali Rana, Usman Hassan, Shaarif Bashir, Mudassar Hussain, Mehroosh Shakeel, Sajid Mushtaq

**Affiliations:** 1 Histopathology, Shaukat Khanum Memorial Cancer Hospital and Research Centre, Lahore, PAK

**Keywords:** adenosarcoma, müllerian, sarcomatous overgrowth

## Abstract

Background and objective

Müllerian adenosarcomas (MA) are rare biphasic tumors with benign epithelial and sarcomatous stromal components. There is very limited cohort study data on MA in the South Asian countries and no such study has been attempted in Pakistan. Our aim was to evaluate the clinicopathological characteristics of MA and to review the published literature on the condition. Additionally, we also analyzed the impact of various prognostic factors on the overall survival (OS) of patients with MA.

Materials and methods

This was a retrospective observational study performed at the Shaukat Khanum Memorial Hospital and Research Centre, Lahore from 2003 to 2020. A total of 59 histologically confirmed cases of MA were included in the study and critically reviewed.

Results

The mean age of the patients was 54 ±16 years, and the most common tumor location was the uterine corpus (48, 81.4%), followed by the cervix (eight, 13.6%), ovary (two, 3.4%), and vagina (one, 1.7%). Sarcomatous overgrowth (SO) was seen in 22 (37.3%) patients, and high-grade cytology was observed in 18 (30.5%) patients. Furthermore, lymphovascular invasion (LVI) was present in six (10.2%) patients, and myometrial invasion was noted in 25 (42.4%) patients. The follow-up details of 29 patients were available, and death was recorded in 13 (44.8%) patients with a median OS of three years.

Conclusion

MA is a rare and diagnostically challenging entity due to its wide differential diagnosis. It is essential to take note of different morphological features such as SO, cytological features, LVI, and heterologous differentiation because of their significant prognostic impact.

## Introduction

Müllerian adenosarcoma (MA) is an uncommon biphasic neoplasm characterized by benign epithelial and malignant stromal components [1]. MA accounts for less than 1% of all female genital tract malignancies and 5% of uterine sarcomas [2,3]. It is most often found in the uterine corpus; however, cervix and extrauterine locations including the vagina, ovary, fallopian tube, retroperitoneum, and urinary bladder have also been described [4-7]. It is believed that MA occurring outside the female genital tract usually arises from preexisting endometriosis [8]. The stromal component usually shows homogenous, low-grade stromal morphology. Sarcomatous overgrowth (SO) is reported in 33-50% of cases [1,9], and a significant proportion of these tumors exhibit high-grade cytology [10]. SO is defined as sarcomatous components occupying at least 25% of the total tumor volume [11]. SO and lymphovascular invasion (LVI) are particularly associated with a higher rate of recurrence and worse survival outcomes [12]. Other prognostic factors include high-grade cytology, myometrial invasion, tumor necrosis, mitotic rate, initial tumor size, and heterologous elements including rhabdomyoblasts [13].

In this study, our aim was to assess clinical and pathological aspects of MA and their impact on prognosis and disease outcomes in our population. To our knowledge, this study is the first of its kind to be conducted in Pakistan and it represents one the largest series of single-center MA cases in South Asia.

## Materials and methods

Approval from the Institutional Review Board (IRB) was obtained prior to the commencement of this study. The hospital information system at the Shaukat Khanum Memorial Cancer Hospital and Research Centre was used to obtain data on verified cases of MA, encompassing the years 2003-2020. A total of 59 cases of MA where slides/blocks and demographic data were available were retrieved. Cases with incomplete histological material or change of diagnosis on review were excluded. The diagnostic material comprised a combination of resection specimens as well as referred blocks/slides from other hospitals.

Clinical information was obtained from the electronic medical records of the hospital information system and through telephone calls. The following clinical parameters were recorded: age of the patients, presenting complaints, treatment, and current status.

Hematoxylin and eosin (H&E)-stained slides of all 59 patients were available. All cases were re-examined by two pathologists. The number of slides ranged from two to 24 (median: four slides). The following gross findings were analyzed: location, morphology, and size of tumors. On microscopic examination, the presence or absence of SO, LVI, high-grade cytology, heterologous elements, and myometrial invasion and mitotic index [mitosis were counted in the hotspot areas in the 10 consecutive high-power fields (HPF)] were recorded. SO was reported when the sarcomatous component of the tumor accounted for 25% or more of the total tumor volume [1]. High-grade cytology was labeled when nuclear atypia and pleomorphism were appreciated at low magnification [8].

## Results

The main clinicopathological and demographic features of MA patients are summarized in Table 1. A total of 59 patients with a mean age of 54 ±16 years were included in the study. Abnormal vaginal bleeding was the most common presenting complaint followed by palpable abdominal mass and protrusion of mass through the cervical os; 47 (79.7%) patients showed polypoidal morphology, whereas 12 (20.3%) patients revealed non-polypoid growth. The most common tumor site was the uterine corpus (48, 81.4%), followed by the cervix (eight, 13.6%), ovary (two, 3.4%), and vagina (one, 1.7%). The mean tumor size was 6.59 ±3.57 cm.

**Table 1:** Baseline clinicopathological characteristics of adenosarcoma patients SD: standard deviation

Variable	Categories	Values
Age (years), mean ±SD		54 ±16
Location of the tumor, n (%)	Endometrium	48 (81.4)
	Cervix	8 (13.6)
	Ovary	2 (3.4)
	Vagina	1 (1.7)
Gross morphology, n (%)	Polypoidal	47 (79.7)
	Non-polypoid	12 (20.3)
Lymphovascular invasion, n (%)	No	53 (89.8)
	Yes	6 (10.2)
Myometrial invasion, n (%)	No	34 (57.6)
	≤50%	17 (28.8)
	>50%	8 (13.6)
Sarcomatous overgrowth, n (%)	No	37 (62.7)
	Yes	22 (37.3)
High-grade cytology, n (%)	No	41 (69.5)
	Yes	18 (30.5)

**Figure 1:**
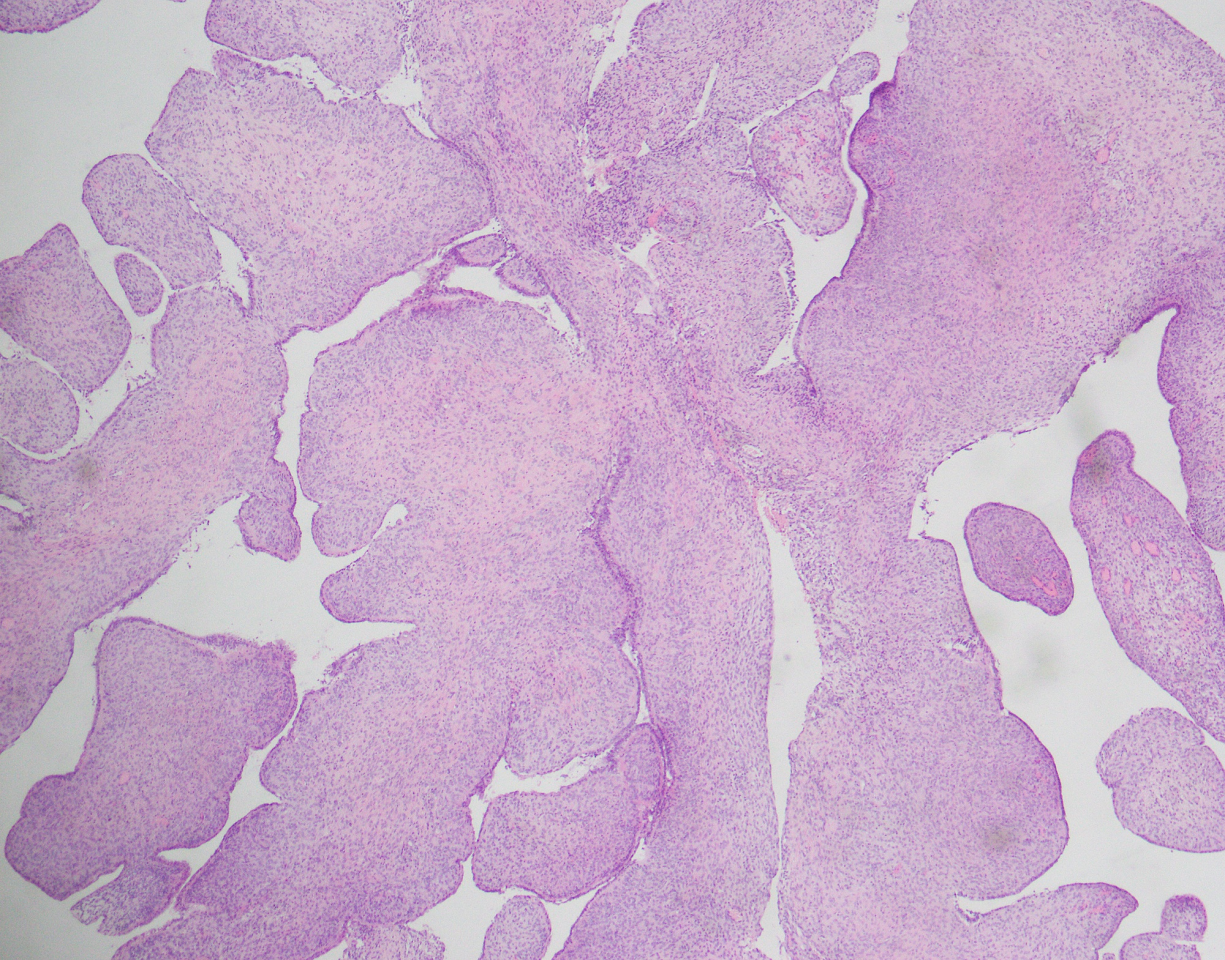
Phyllodes-like histological pattern of adenosarcoma (H&E 10X) H&E: hematoxylin and eosin

**Figure 2:**
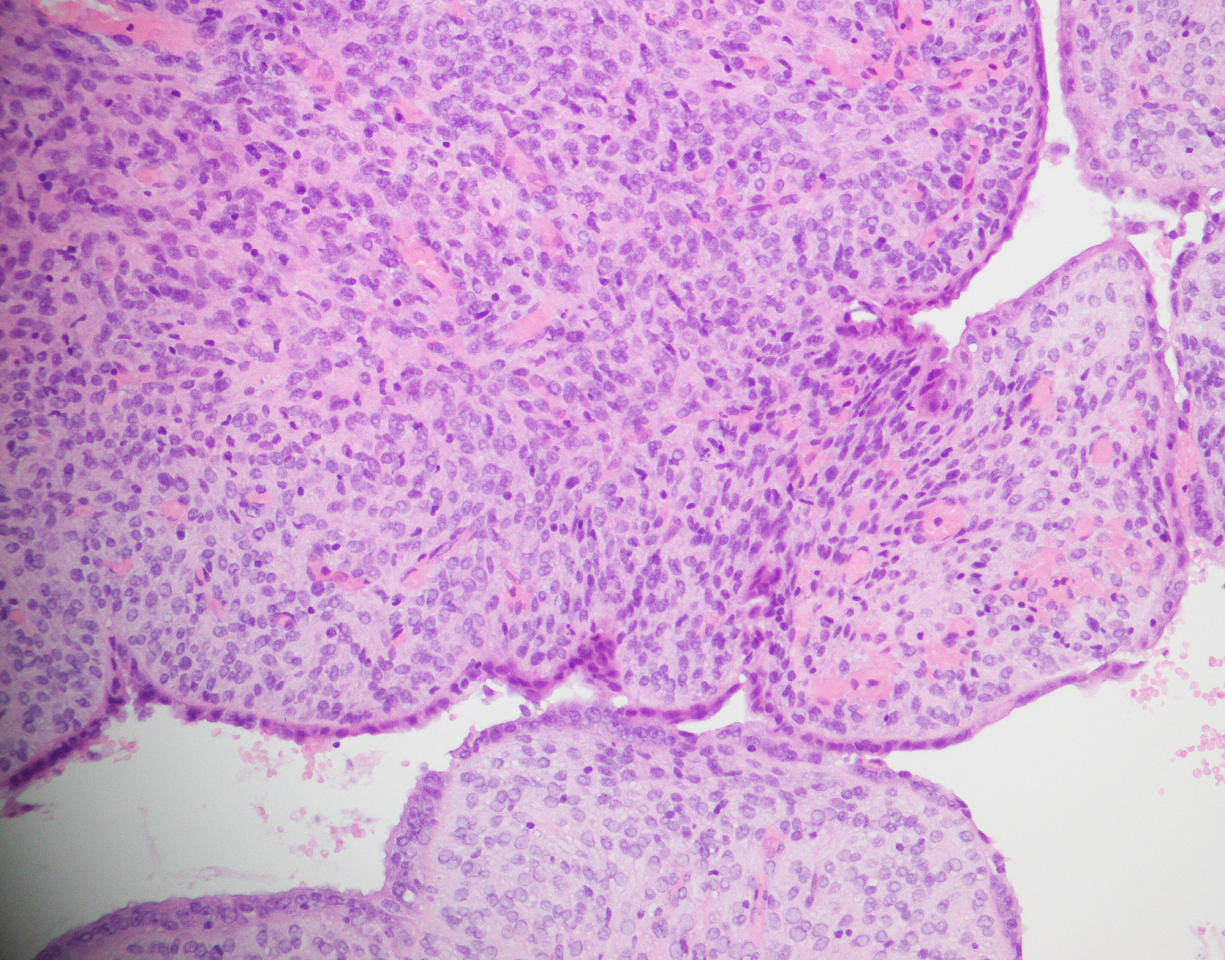
Sheets of oval to spindle mildly atypical cells with periglandular stromal condensation (H&E 20X) H&E: hematoxylin and eosin

Histopathological examination revealed the majority of the tumors showing phyllodes-like growth pattern (Figure 1, Figure 2). Stromal condensation was seen in periglandular areas. The epithelium was of endometroid type in 56 cases. Among these, squamous and mucinous metaplasia was noted in eight cases. The endocervical-type epithelium was seen in two cases and squamous epithelium was noted in one case.

No SO was observed in 37 (62.7%) cases. All these tumors displayed low-grade mesenchymal component with the morphology of predominantly low-grade endometrial stromal sarcoma. Focal smooth muscle differentiation was present in seven cases [highlighted by smooth muscle actin (SMA) and desmin]. Mitotic index was 2-23 per 10 HPF with a median of five mitoses per 10 HPF.

**Figure 3:**
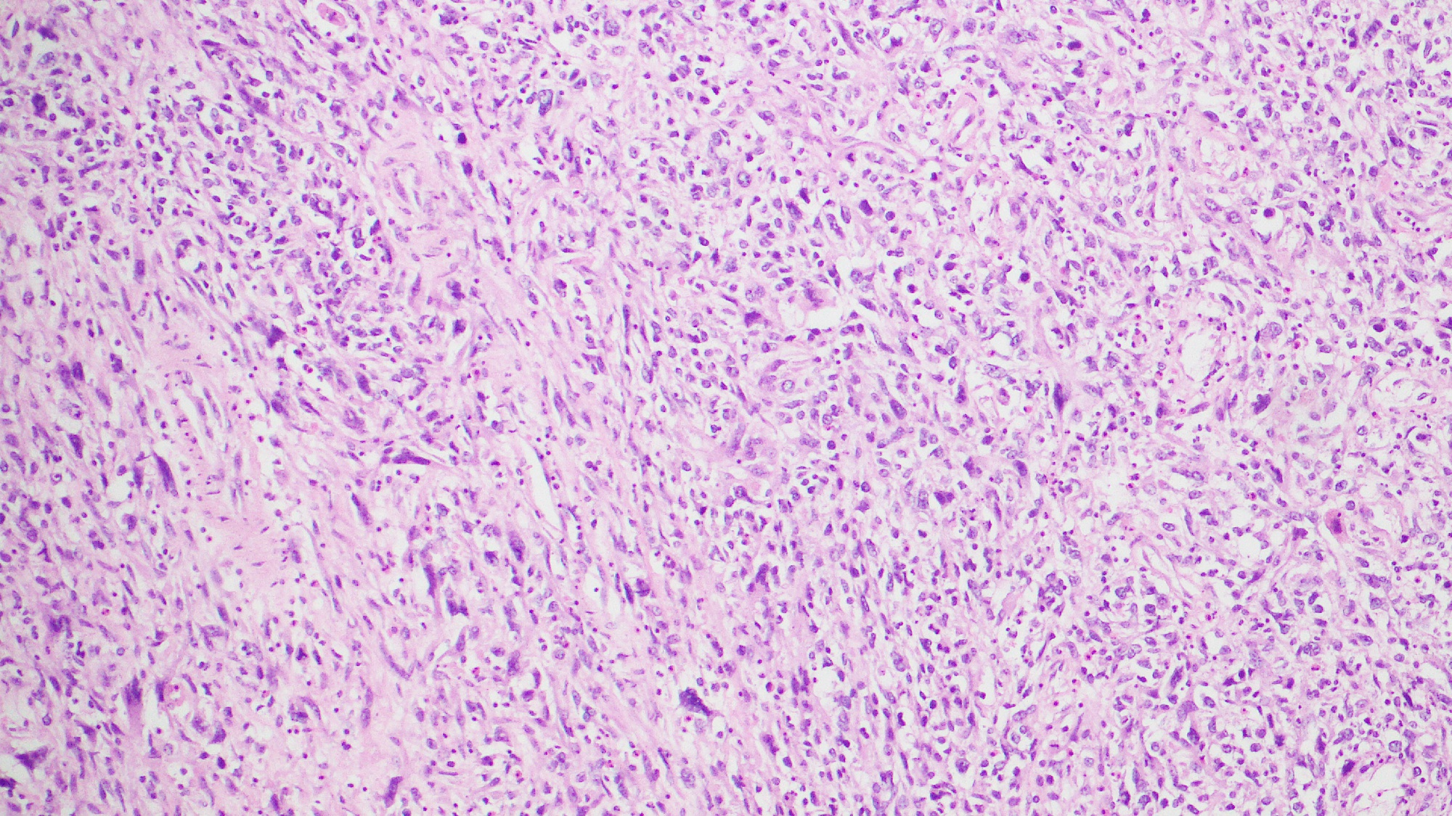
Cytological atypia (H&E 20X) H&E: hematoxylin and eosin

**Figure 4:**
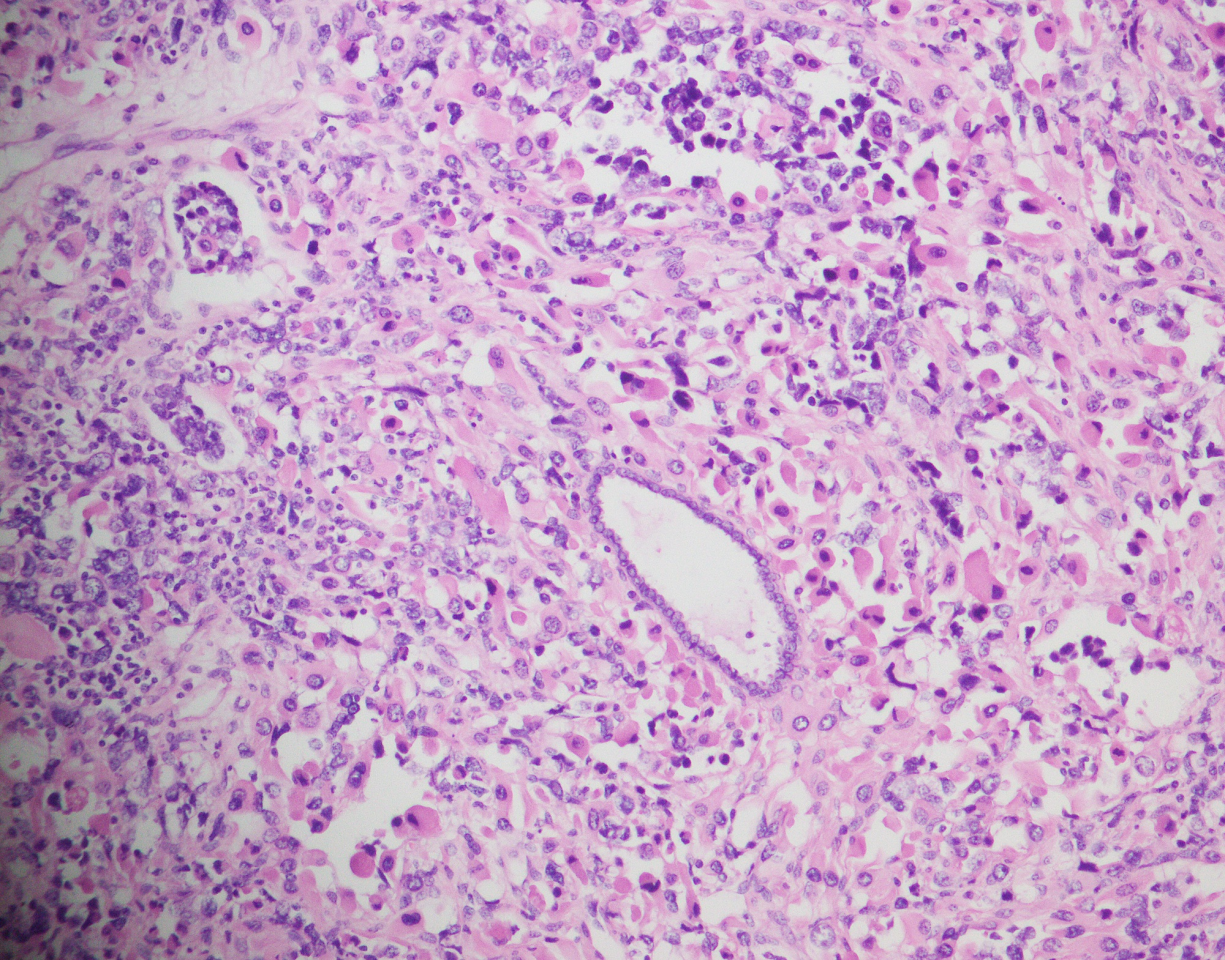
Rhabdomyosarcomatous differentiation (H&E 40X) H&E: hematoxylin and eosin

SO was seen in 22 (37.3%) cases and high-grade cytology was present in 18 of these cases (Figure 3). Sarcomatous component was high-grade endometrial stromal sarcoma in 17 cases and leiomyosarcoma in five cases. Fibrosarcoma-like areas were seen in two cases, and heterologous mesenchymal element was seen in eight cases. Among these, embryonal rhabdomyosarcoma-like differentiation was noted in seven cases (Figure 4) and cartilaginous differentiation in one case. Mitotic index was 7-52 per 10 HPF (median: nine mitoses/10 HPF). LVI was present in six (10.2%) patients and myometrial invasion was seen in 25 (42.4%) patients [≤50% in 17 (28.8%) patients and >50% in eight (13.6%)] respectively (Table 1).

**Figure 5:**
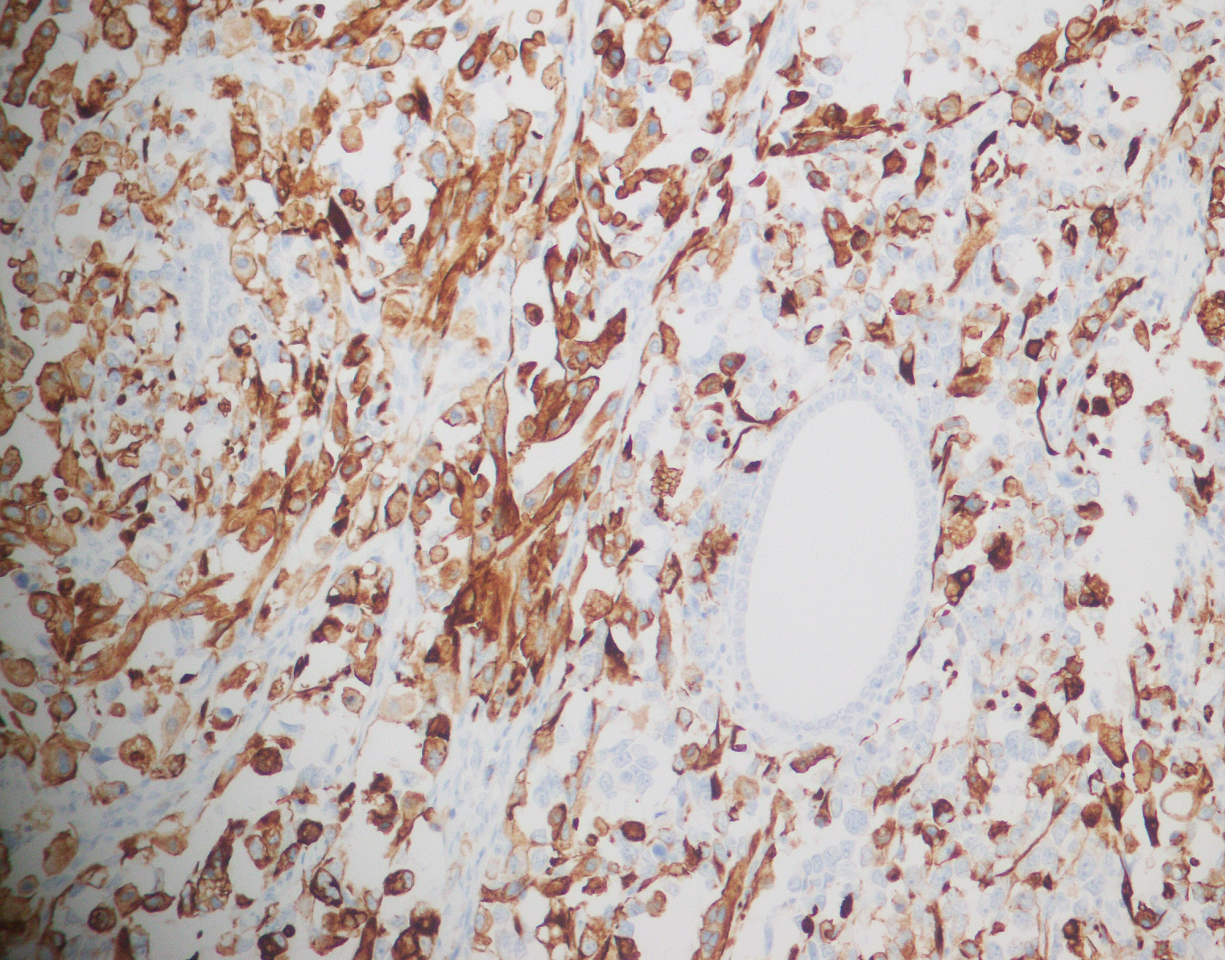
Desmin immunohistochemical stain (40X)

**Figure 6:**
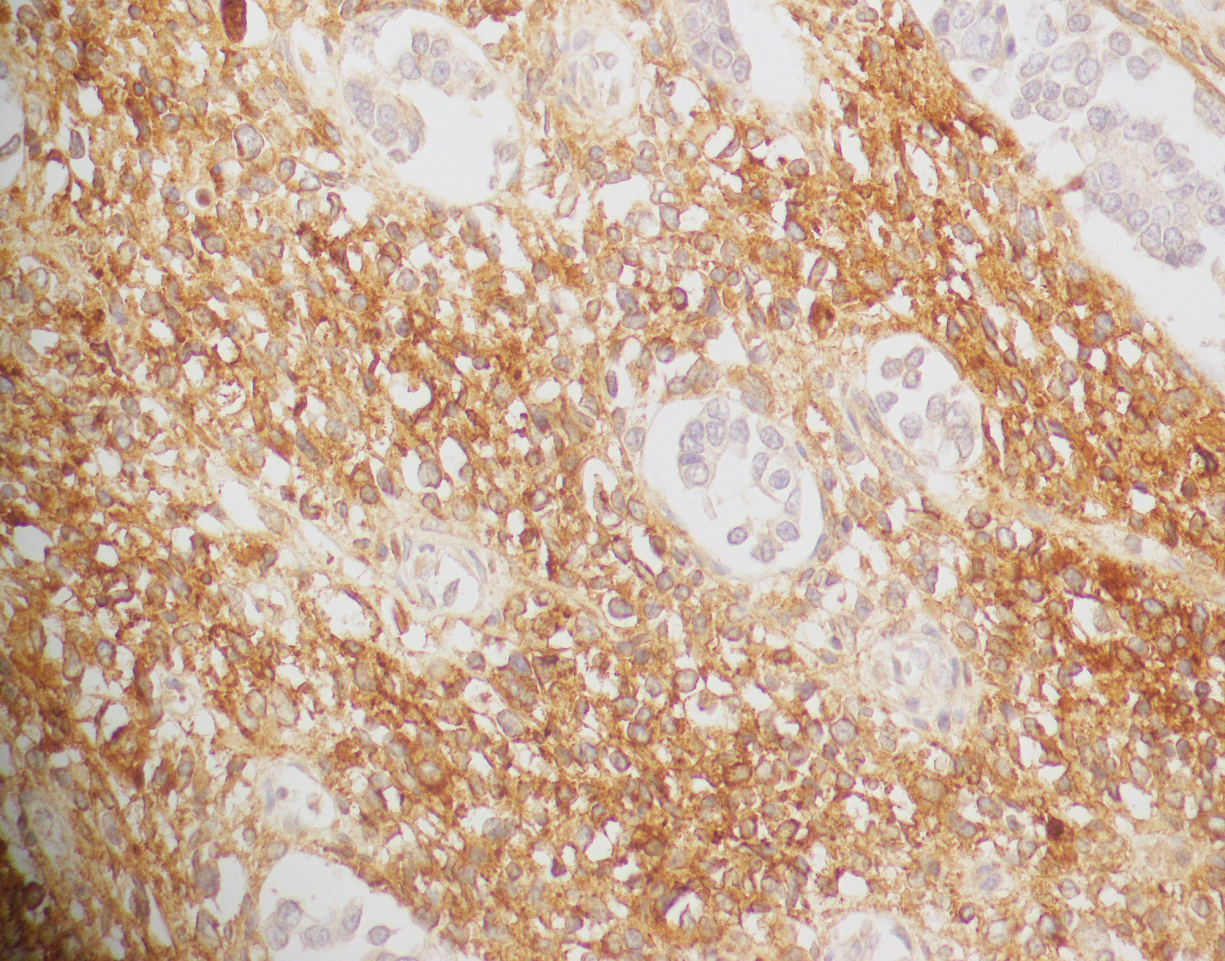
CD10 immunohistochemical stain (40X)

In patients in whom immunohistochemical stains were performed, the positive rate of desmin was 10/14 (70.4%), and that of CD10 was eight out of eight (100%). (Figure 5, Figure 6). Cytokeratin was performed in 23 cases, and focal expression in the stromal component was observed in two cases. Likewise, p53 was performed in six cases and staining was of wild type in all cases. Similarly, estrogen receptor (ER) and progesterone receptor (PR) were performed in three cases, and no staining was seen in any of these. Ki67 showed variable proliferation index (median: 19, range: 2-80%).

Limited follow-up data of 29 patients were available. Of these, 19 (65.6%) patients received adjuvant therapy, and four (21.0%) patients received radiotherapy only; 15 of the 19 (79.0%) patients who received adjuvant therapy received both chemotherapy and radiotherapy. Local recurrence was noted in two (6.9%) patients, whereas death was recorded in 13 (44.8%) patients. The percentage of patients who died of disease (DOD) was 61.5% (8/13), as shown in Table 2.

**Table 2:** Follow-up clinical information of adenosarcoma patients

Variable	Categories	Values, n (%)
			Adjuvant therapy	No	10 (34.4)
	Yes	19 (65.6)
	Radiotherapy	4 (21.0)
	Chemotherapy + radiotherapy	15 (79.0)
Recurrence	No	27 (93.1)
	Yes	2 (6.9)
Status	Alive	16 (55.2)
	Deceased	13 (44.8)
	Cause of death	
	Primary disease	8 (61.5)
	Other	5 (38.5)

Of the eight patients who DOD, six (75%) had SO with four of them having >50% myometrial invasion and two having a local recurrence. The remaining two (25%) patients did not have a myometrial invasion or high-grade cytology; however, both exhibited LVI. No documented recurrence was present in these two cases. Further follow-up analysis of the patients is summarized in Table 3.

**Table 3:** Bivariate analysis of pathological characteristics of adenosarcoma patients SD: standard deviation

Variables	Categories	Alive (16, 55.2%)	Deceased (13, 44.8%)	P-value
Age (years), mean ±SD		53.56 ±14.03	64.54 ±5.07	0.01
Size of the tumor (cm), mean ±SD		6.50 ±3.77	9.15 ±3.80	
Location, n (%)	Cervix	3 (18.8)	2 (15.4)	1.00
	Endometrium	12 (75.0)	11 (84.6)	
	Right ovary	1 (6.2)	0 (0.0)	
Gross morphology, n (%)	Non-polypoid	1 (6.2)	2 (15.4)	0.57
	Polypoid	15 (93.8)	11 (84.6)	
Lymphovascular invasion, n (%)	Absent	16 (100.0)	10 (76.9)	0.07
	Present	0 (0.0)	3 (23.1)	
Sarcomatous overgrowth, n (%)	Absent	12 (75.0)	6 (46.2)	0.14
	Present	4 (25.0)	7 (53.8)	
High-grade cytology, n (%)	Absent	12 (75.0)	6 (46.2)	0.14
	Present	4 (25.0)	7 (53.8)	
Myometrial invasion, n (%)	No	13 (81.2)	7 (53.8)	0.19
	≤50	2 (12.5)	2 (15.4)	
	>50	1 (6.2)	4 (30.8)	
Rhabdomyoblastic differentiation, n (%)	Absent	16 (100.0)	8 (41.5)	0.01
	Present	0 (0.0)	5 (38.5)	
Recurrence, n (%)	No	16 (100.0)	11 (84.6)	0.19
	Yes	0 (0.0)	2 (15.4)	

**Figure 7:**
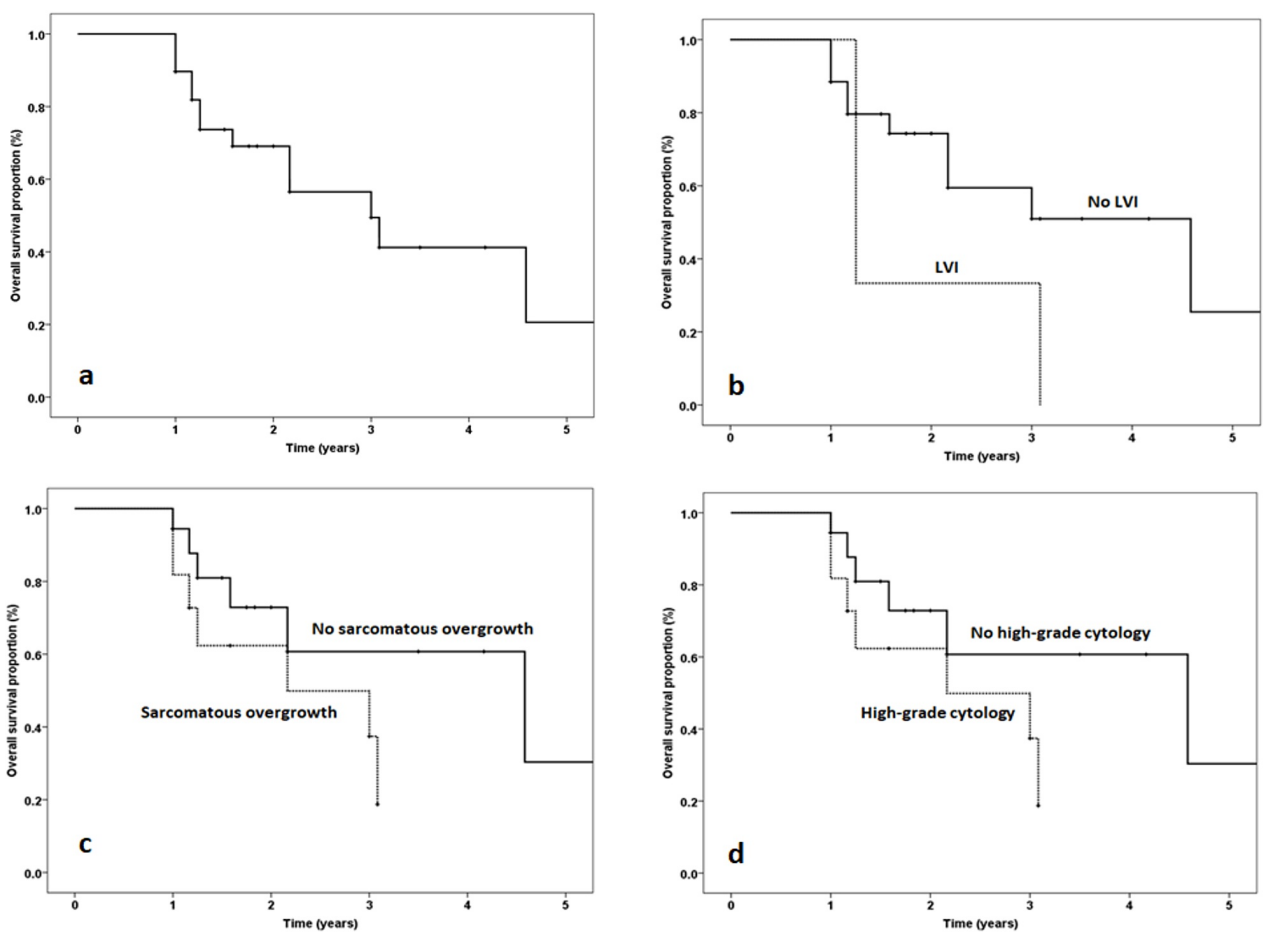
Kaplan-Meier curves for median overall survival in patients with uterine adenosarcoma and those with and without LVI, SO, and high-grade cytology Kaplan-Meier curves: a) median overall survival in patients with uterine adenosarcoma; b) with and without LVI; c) with and without sarcomatous overgrowth; d) with and without high-grade cytology LVI: lymphovascular invasion; SO: sarcomatous overgrowth

The median overall survival (OS) of the patients was three years (Figure 7a). LVI was associated with decreased OS (1.25 years vs. 4.6 years) (Figure 7b). Similarly, SO and high-grade cytology were associated with poor disease outcome (median OS: 2.6 years) as compared to those cases with none of these features (median OS: four years) (Figure 7c, Figure 7d). Furthermore, the three-year disease-specific survival (DSS) was 62%, and rhabdomyosarcomatous differentiation and LVI were associated with poor DSS.

## Discussion

MA was first reported by Clement and Scully [14] in 1974. These are generally low-grade tumors [15]. Leaf-like patterns, periglandular cuffing, and rigid cystic glands are the characteristic morphological features [16]. Its differential diagnosis includes both benign and malignant entities. Benign entities are endometrial polyp, adenomyomatous polyp, endocervical polyp, and adenofibroma. MA was differentiated from these entities based on the generally larger size of these tumors, absence of prominent central blood vessels, and presence of typical architecture, periglandular stromal condensation, and increased mitosis. In a few difficult cases, Ki67 has been performed, which showed increased proliferation in periglandular areas. The malignant entities include endometrial stromal sarcoma, malignant mixed Müllerian tumor (MMMT), and leiomyosarcoma [17,18]. MA is distinguished from endometrial stromal sarcoma by the all-round distribution of glands, presence of characteristic leaf-like pattern, and condensed stromal areas around glands. Likewise, the presence of the benign epithelial component helps in establishing the diagnosis of MA and can exclude MMMT. When in the cervix, botryoid rhabdomyosarcoma (especially in younger patients) is considered in the differential diagnosis; however, the absence of staining for desmin and myogenin helps to reach the correct diagnosis of MA.

Gallardo and Prat [1] studied 55 cases of MA in 2009. In their study, the mean age of the patients was 59 years; 48 tumors (87.2%) were located in the uterine corpus and cervix (37 in endometrium and 11 in the cervix), 19 and seven tumors were located outside of the uterus and cervix, respectively. The majority of the patients in their study had initial complaints of abnormal vaginal bleeding and pain in the abdomen. The mean size of the tumor was 6.5 cm. In our study, patients' mean age was 54 ±16 years. In 56 patients, MA was located in the uterine corpus and cervix [48 (81.4%) patients had tumors in the endometrium and eight (13.6%) in the cervix]. Extrauterine location was also observed in three cases (in the ovary for two cases and vagina in one case). The mean tumor size was 6.59 ±3.57 cm. These findings show concordance of our study with the above-mentioned study in terms of age of patients, tumor size, and tumor location.

A review of the literature has shown that the percentage of SO in MA ranges from 8% to 54% [1,19]. In a study conducted by Gallardo and Prat. (2009) [1], SO was seen in 18 (33%) cases and mitotic index varied from less than one to 30 per 10 HPF (mean: 3/10 HPF). Embryonal rhabdomyosarcoma was noted in 10 out of 55 cases. Likewise, another study conducted by Yuan et al. (2019) [2] revealed that 15 (13.6%) patients showed SO, four (8.2%) patients had heterologous elements, two patients showed areas resembling embryonal rhabdomyosarcoma, six patients had LVI, and five patients showed tumor necrosis. In our study, SO was seen in 22 (37.3%) patients, and heterologous elements were seen in eight cases, out of which seven patients had a rhabdomyosarcoma component. Necrosis was observed in 12 cases, and LVI was noted in six (10.2%) cases. These findings are compatible with the above-mentioned Western studies.

Prognostic factors of MA include SO, LVI, high-grade cytology, heterologous elements, higher disease stage, and advanced age [10,20-22]. Carrol et al. [19] studied 74 patients of MA in 2014. They postulated that SO and LVI are poor prognostic factors, and these patients have a significantly lower OS rate of 4.61 years. In our study, we also looked into the impacts of SO, LVI, high-grade cytology, and heterologous elements on the survival of the patients. We found that LVI is associated with poor survival (median OS: 1.25 years). Similarly, the median OS was 2.20 years for both SO and high-grade cytology. In line with other studies [23,24], we concluded that the dismal disease outcome was associated with the presence of rhabdomyosarcomatous elements (median DSS: 1.17 years). Although these findings are in agreement with other Asian [21,22] and Western studies [3-7] regarding poor prognostic factors, the median OS was much lower in our population compared to the patients in the developed countries. This can be attributed to poor healthcare services, overall poor nutritional status, limited diagnostic facilities, insufficient knowledge of the disease, inconsistent follow-up, and lack of standardized treatment protocols in a Third World country like Pakistan.

## Conclusions

MA is characterized by diverse clinical and pathological features. It is important to record pathological parameters such as SO, high-grade cytology, LVI, and rhabdomyosarcomatous differentiation in patients as they can adversely affect disease outcomes.
